# Treatment Adherence in Chronic Conditions during Ageing: Uses, Functionalities, and Cultural Adaptation of the Assistant on Care and Health Offline (ACHO) in Rural Areas

**DOI:** 10.3390/jpm11030173

**Published:** 2021-03-02

**Authors:** David Conde-Caballero, Borja Rivero-Jiménez, Carmen Cipriano-Crespo, Manuel Jesus-Azabal, Jose Garcia-Alonso, Lorenzo Mariano-Juárez

**Affiliations:** 1 Department of Nursing, Faculty of Nursing and Occupational Therapy, University of Extremadura, 10003 Cáceres, Spain; dcondecab@unex.es (D.C.-C.); lorenmariano@unex.es (L.M.-J.); 2 Department of Computer and Telematic Systems Engineering, Polytechnic School, University of Extremadura, 10003 Cáceres, Spain; manuel@unex.es (M.J.-A.); jgaralo@unex.es (J.G.-A.); 3 Department of Nursing, Physiotherapy and Occupational Therapy, Faculty of Health Sciences, University of Castilla La Mancha, 13071 Ciudad Real, Spain; Mariacarmen.Cipriano@uclm.es

**Keywords:** treatment adherence, chronically ill patients, voice assistant, rural, ageing

## Abstract

The increasingly common scenario of an ageing population is related to a rise in the prevalence of problems associated with chronic conditions and comorbidities. Polypharmacy is frequent among this population, and it is a situation that can create medication management and adherence issues. This article introduces the features and functionalities of a voice assistant (Assistant on Health and Care Offline, ACHO) that aims to facilitate treatment adherence among elderly adults. Specifically adapted for its use in rural contexts, it does not require an Internet connection. Its development consisted of two stages: a first stage of problem diagnosis, in which the classic tools of ethnographic fieldwork were used, and a second stage of design implementing methodologies developed by Ambient Assisted Living (AAL) programmes. The main design characteristic of this new digital care system is that it is adapted to the needs of its end-users. It includes features such as voice customisation and the personal identification of medication, it can be connected to other digital devices, and information is introduced and supervised by healthcare professionals. These custom features introduce a safer medication administration procedure, improve supervision strategies, and increase patients’ trust in the prescription process.

## 1. Chronicity and Treatment Adherence in Ageing

The global population, and particularly that of Western countries, is ageing rapidly. According to data from 2018, almost one-fifth of the European population (19.7%) was 65 years of age or older [1]. In some countries, such as Spain, the situation is even more dramatic. In the four decades between 1978 and 2018, the ageing index in this country has increased from 37.3 to 120.5 [2]. According to current projections, by 2068, the percentage of elderly population will be 29.4% [3]. This phenomenon can be included within the so-called “second demographic transition” [4,5], which is defined by a decrease in childhood mortality and an improvement in lifestyles that has led to an increased life expectancy among the elderly [6]. The World Health Organisation (WHO) has pointed out that this scenario poses a major public health challenge for the 21st century [7]. The increase in the percentage of the population living with complex age-related illnesses, the financial burden of providing quality healthcare, or the sustainability of healthcare systems are just a few of the issues that need to be addressed in the short term [8,9].

One of the consequences of the rapid growth of ageing populations is the rise in the prevalence of chronic illnesses and comorbidities [10,11,12]. In turn, this magnifies the problem of treatment adherence among elderly individuals—particularly for those that have been prescribed multiple medications [13,14]. The term “polypharmacy” has been suggested for patients taking more than five different medications on a daily basis [15]. Paci et al. [16] noted that 40% of patients between 75 and 84 years of age examined in their study had been prescribed more than 10 different medications—a percentage that can be even higher among rural, elderly populations [17,18,19,20]. Improving treatment adherence among the elderly can be particularly challenging due to several associated issues—i.e., a higher risk of adverse drug events, difficulties opening tubs of medications or swallowing larger pills, lack of understanding about their treatment, lack of awareness about their illness, or memory loss—all of which can be even worse for patients living alone. Although measuring rates of treatment adherence presents its own difficulties [21,22,23], studies such as that of Carrillo et al. [24] noted that a third of elderly, chronically ill patients failed to follow their prescribed treatment correctly—a percentage similar to that suggested by Yilmaz and Colak [25]. The WHO [26] considers that this percentage is more likely around 50%, which is similar to the 51.7% some authors have suggested for the Spanish context [27].

Proposals for improving treatment adherence have been suggested from a variety of perspectives and scientific disciplines. For instance, a number of projects have focused on reinforcing the patients’ capacity and willingness to follow treatment by supporting their social and family environment [28]. Others have highlighted the importance of improving communication between healthcare providers and patients [29]. Different technologies and devices have been developed to help with these issues. Technological innovations have played a major role in the development of e-Healthcare, and they are particularly relevant in the case of treatment adherence [30]. Whereas in the past patients relied on different recall techniques such as notes left in different rooms, alarm watches, or pillboxes with appropriate dosages, nowadays, improvements in connectivity and increased Internet access have opened new avenues—with the possibility of instant communication, updates, and interactions. Electronic medication packaging devices [31], the Medication Event Monitoring System (MEMS^®^) (Aardex Group, Fremont, CA, USA) [32], the Turbo-inhaler computer^®^ (Astra Draco, Lund, SWE), Doser^®^ (Meditrack Products, S. Easton, MA, USA), Cerepak^®^ (Information Mediay Corp., Ottawa, ON, Canada, YOW, CAN), or Dosepak^®^ (WestRock, Atlanta, GA, USA) are a few examples of currently available electronic devices [33].

However, the problem with current trends in e-Healthcare, Ambient Assisted Living (AAL), and robotic technology is their dependence on digital connectivity. Consequently, patients without Internet access remain largely excluded from their reach. The objective of this article is to present the Assistant on Care and Health Offline (ACHO), which aims to fill this gap—a Bluetooth voice assistant whose use does not require an Internet connection, specifically conceived for elderly adults, and whose design is based on an interdisciplinary research project.

## 2. Materials and Methods

### 2.1. Study Design

ACHO was developed through the collaboration of an interdisciplinary group of computer scientists, nurses, and anthropologists within the International Institute for Research and Innovation on Ageing (4IE+). This collaborative project, developed by Spanish and Portuguese researchers, aimed to improve knowledge on issues related to the health and well-being of elderly, rural populations, and to develop technical solutions to address specific problems. Therefore, the project consisted of two main stages:

The first stage of diagnosis was based on ethnographic fieldwork [34,35,36]. Through different qualitative research tools and techniques, relationships were established with the study subjects in their everyday contexts—i.e., their homes and villages. The aim of this approach was to understand the study subjects’ own definitions and experiences, and their perception of the main health-related issues affecting them. Fieldwork focused specifically on assessing the social practices related to the management of prescribed medications, in particular patients’ strategies to adhere to the treatments prescribed and problems arising in relation to this. 

The second stage of the project, design, was based on the analysis of the empirical material collected and aimed to provide solutions to the issues identified. This approach is based on the methodology developed by Ambient Assisted Living programs [30,37], with interdisciplinary research network focused on developing technological solutions—and particularly interested in the new possibilities presented by context-adapted voice assistants.

### 2.2. Ethics Approval

This research project was approved by the Bioethics and Biosafety Committee of the University of Extremadura (Ref. 143/2020). All participants signed a consent form whereby they were informed of the purpose of their participation in the study. Participants were also informed that they could discontinue their participation at any time without further consequences. The confidentiality of personal data was guaranteed throughout the research process, which is in line with current Spanish law (Organic Law 15/2019, of 13 December, on Protection of Personal Data). One member of the research team anonymised the participants’ personal data before the analysis of material collected during the first stage of diagnosis, and the rest of the team only had access to coded identities. The study was conducted in accordance with the ethical principles outlined in the Declaration of Helsinki [38] and the Belmont Report [39].

### 2.3. Participants

The fieldwork was carried out in several localities in the Autonomous Community of Extremadura (Spain). Based on official statistics [40], the population of Extremadura was estimated at 1,065,575 in 2020 (2.23% of the total Spanish population). Its population density is very low—26 inhabitants per square kilometre, while the Spanish national average is 92 inhabitants per square kilometre. The percentage of elderly population in this region was estimated at 20.64% in 2019, with an ageing index of 140.84 and a dependency ratio of 54.56—all above the national average. 

Observational units were established in the locations chosen for fieldwork, followed by a non-probability sampling of older adults to be interviewed on topics related to their health and well-being, in order to obtain a variety of perspectives [41]. Interviews and participant observation in homes were carried out on 34 participants. Criteria for inclusion were as follows:

- Over 65 years of age.
- Non-institutionalised (in care homes, day-care centres, or any other kind of facility).
- Not subject to cognitive decline of any kind.
- To have been prescribed at least one chronic illness medication by a doctor.
- To have voluntarily accepted participation in the study and provided informed consent.

### 2.4. Data Collection and Measures

Fieldwork to collect data and empirical materials—including participant observation and in-depth, semi-structured interviews (Table 1)—was conducted between March 2019 and January 2020. Topics emerging during informal conversations were also used in the definition of interview categories [42,43], and later on in the design of specific functionalities for the voice assistant. Finally, a field diary provided additional information on specific contexts and topics, helping establish a more complete picture of the participants’ experiences and needs—as observed in research projects previously conducted [44].

#### 2.4.1. Participant Observation

Participant observation has been defined as a form of production of knowledge through “being and action”—through their active presence and engagement in the environment studied, researchers are attuned to registering activities and particularities that could be relevant for their research [45,46]. Members of the research team observed everyday life and self-care practices among the participants, in their own homes and through their everyday activities in their villages—in a variety of places such as healthcare centres, pharmacies, and public social spaces such as bars or leisure centres for the elderly. Practices noticed through participant observation—such as the habit of “hoarding” medications or giving them alternative names to avoid confusion—were later translated into specific functionalities in the design stage.

#### 2.4.2. Semi-Structured Interviews

The main source of data was in-depth, semi-structured interviews [47,48]. These were conducted by a team of three researchers with extensive experience in qualitative research and in-depth interviews. The interviews followed a structured guide, containing subject areas and theoretical questions, which were developed based on a review of literature on similar studies. One of our main goals was to explore the development of what are known as “therapeutic itineraries” [49,50,51]: patients’ personal practices and representations associated with a medical condition and the actions that they undertake while seeking a solution to these. Our study focused particularly on potential problems arising while trying to comply with medical prescriptions and the study participants’ adherence to the treatments they had been prescribed. Some examples of questions are included in the following table (Table 2).

#### 2.4.3. Field Diary

The field diary was established as one of the researchers’ fundamental tools since the first works of classical anthropology [52]. The field diary is a secondary, systematic, reflective, and intelligible record of what happened during the day in the field [53]. It contains notes taken during fieldwork, based on observations, informal conversations, and interviews, but it also adds some reflection. It provides contextual and other information. In this project, notes were collected in a field diary shared by the researchers who participated in the fieldwork. These fieldnotes were related to various aspects of direct participant observation. They were transcribed, coded, and analysed together with the rest of the empirical material.

### 2.5. Data Analysis

All the empirical material collected through different research techniques (participant observation, semi-structured interviews, informal conversations, and fieldnotes) was coded for content analysis. One of the members of the research team coded the initial categories of analysis, following an inductive–deductive approach [54,55]. This approach, in addition to the verification of hypotheses relevant to the context analysed (i.e., previously reviewed technological features and solutions—deductive approach), is open to the generation of new hypotheses (i.e., new culturally adapted technological features and solutions such as the customisation of medication names, which was one of the strategies identified for treatment adherence—inductive approach).

Then two researchers examined, analysed, and re-interpreted the empirical materials, while the third one arbitrated in their discrepancies, thus completing the triangulation. The whole of the research team (anthropologists, health professionals, and computer scientists) analysed together the categories established, using the qualitative data analysis software ATLAS.ti (Scientific Software Development GmbH, Berlin, Germany, version 8.4.24.0 for Windows). It has been suggested that collaboration between professionals with different backgrounds and skills is crucial for a research projects such as ours to succeed [56]. Our interdisciplinary approach brings an anthropological perspective into that of the computer scientists’, which is more focused on problems/solutions. It incorporates the perspective of sociological imagination [57], while also paying attention to the uniqueness of each context and the diversity of social life in rural areas. The resulting proposal is a voice assistant designed to remind patients when their medical appointments or medications are due and adapted to the specific needs of ageing populations living in rural contexts.

## 3. Results

In recent years, there has been increasing interest in the promotion of home-based care [58,59]. The WHO defines this as any form of care or assistance provided to vulnerable or elderly populations in their homes by informal caregivers (i.e., relatives, friends, local community members), in cooperation with trained healthcare professionals. This option is preferred both by patients and their relatives, and it is also more cost-efficient for healthcare providers than institutionalisation in care facilities [59,60]. Home-based care is in line with WHO guidelines as expressed in the International Classification of Functioning, Disability and Health (ICF) [61]—which emphasise the importance of supporting older adults to retain their functional capabilities in ageing while remaining in their preferred environments for as long as possible. Following this viewpoint, and with the aim of improving treatment adherence among elderly adults living in rural areas, we present here a first version of a voice assistant based on open-source platforms—whose scope and functionalities are based on needs identified through fieldwork.

**Figure 1:**
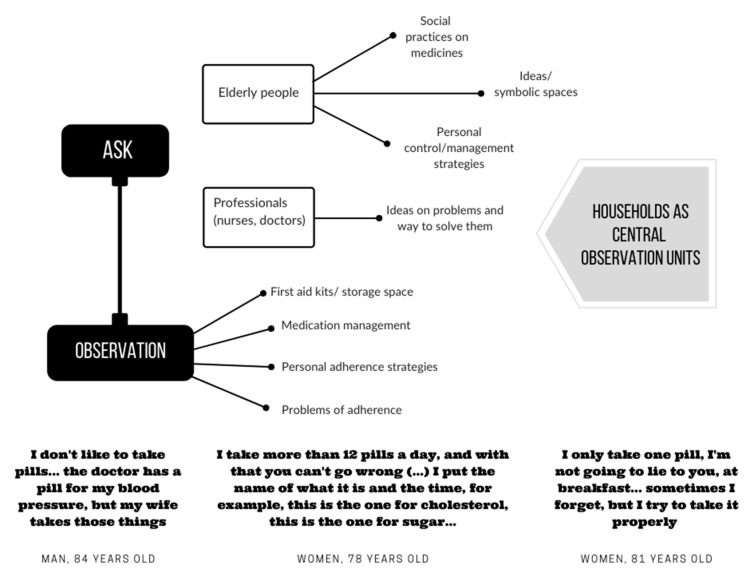
Qualitative data results.

The results of the qualitative research have been instrumental in the development of ACHO. Some of these findings are explained in Figure 1. For example, the main findings from observation pointed to the need for older people to have support in remembering to take medication. This was reflected in the observation in several of the households of different notes placed in the medicine cabinets or on magnets on the fridges, which helped older people remember how many and when they had to take their medicines. As for the main findings of the interviews, we discovered that many of the participants personalised the names of the pills. For example, they called the blood pressure pill by the colour of the pill or “the one I take with food”.

### 3.1. ACHO Design

**Figure 2:**
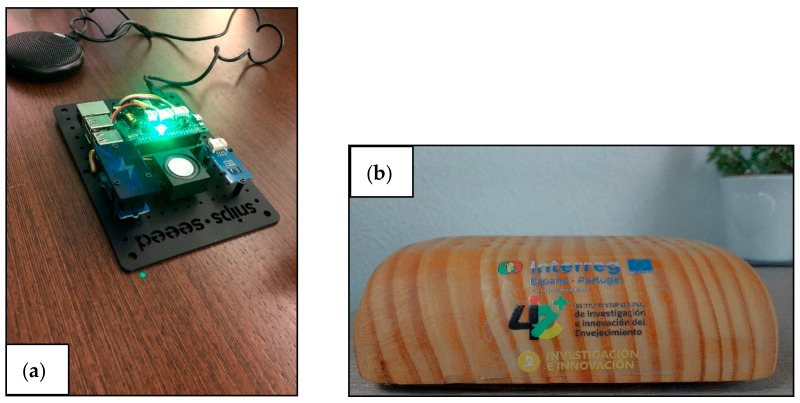
Current appearance of Assistant on Health and Care Offline (ACHO): (**a**) internal and (**b**) external.

This voice assistant (Figure 2), which we have named Assistant on Care and Health Offline (ACHO), uses a Snips-type voice platform [62] to create an interface adapted to the context and specific needs of the elderly population studied. The main feature of this open-source platform is that it is not cloud-based and it does not require an Internet connection—all information is kept on-device. This allows ACHO to be used in any household and region, independently of the range of Internet connectivity and coverage (3G/4G) in the area. It is important to recognise that many elderly adults do not have Internet access for different reasons—lack of interest, low levels of computer literacy, low income, or lack of adequate connectivity in rural areas, among others.

### 3.2. Operation

The first version of ACHO was developed to remind users when medication and medical appointments were due [63]. The device was programmed to proactively create a notification at a certain time and date, when an event was due. This functionality was intended to improve treatment adherence among elderly patients, while also supervising and monitoring their medication schedule. Additionally, the assistant could perform checks on the overall health of the user through a series of questions, registering the interaction and answers received—thus creating two-way datasets that could be analysed by healthcare professionals at a later stage.

Among the assistant’s current features is the possibility of customising the medication schedule for each user—re-naming each medication the way the patient prefers and not by its commercial or generic name, which can help avoid confusion (i.e., “the cholesterol pill”, “the pill from the blue box”). The operation of the device involves two different components: a mobile application and the voice assistant itself.

#### 3.2.1. Mobile Application

**Figure 3:**
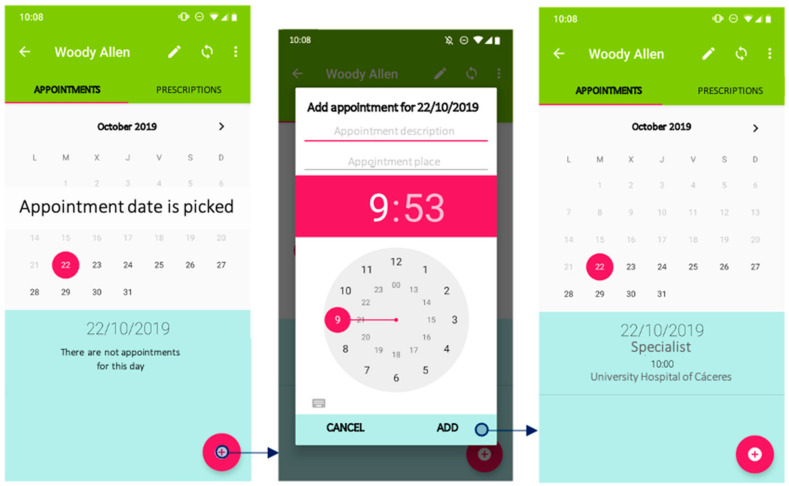
Creating a new medical appointment with the mobile application.

**Figure 4:**
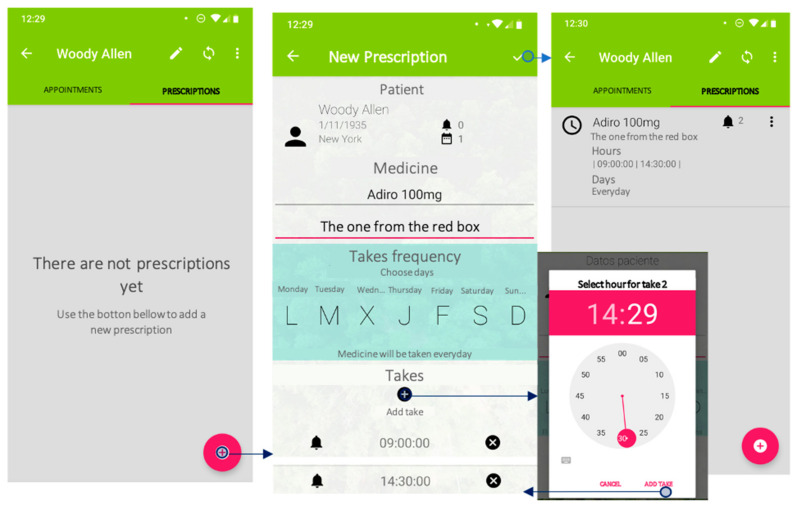
Adding a new prescription with the mobile application.

Information on the patient, including medical appointments and prescriptions, is introduced through a mobile application. This application provides an entry point for data input directly from a smartphone. The application can store different patients’ profiles, and the different options can be easily and smoothly navigated (Figure 3). Its ease of use makes it accessible for any user, facilitating interaction and queries. The main purpose of this application is to allow healthcare professionals and caregivers to introduce information even if not familiar with mobile applications—since this is a safer medication administration procedure (Figure 4). These “digital” care prescriptions would be monitored by community nurses, who would synchronise and review the data logged in the device during their home visits. Therefore, data are always kept at a local, on-device level. Data synchronisation could also be undertaken by a relative.

**Figure 5:**
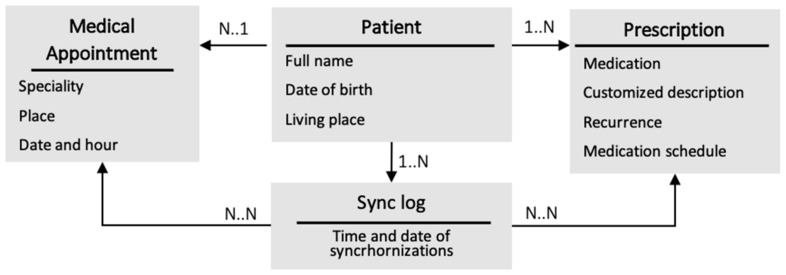
Components of the mobile application database.

The voice assistant is ready for synchronisation once the profile has been completed, with the introduction of new appointments and prescriptions in the mobile application following the scheme detailed in Figure 5. Since the idea was for the smart speaker to operate with no Internet connection, it was necessary to introduce specific functionalities that guaranteed that information was readily available at any time.

**Figure 6:**
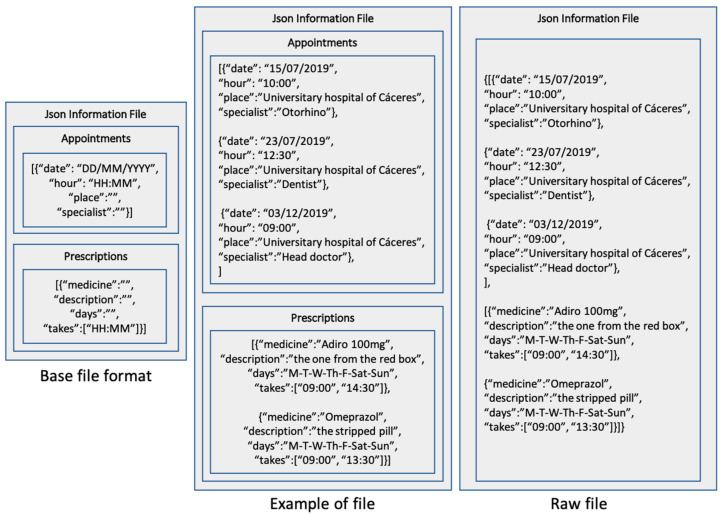
Format of the file generated by the application.

The process of patient data input and storage of information ends with the synchronisation of the ACHO voice assistant. The device does not require an Internet connection, using instead Bluetooth technology for synchronisation. To start the process of synchronisation, only physical proximity to the smart speaker is required, once the corresponding option has been selected in the mobile application. Internally, the device then generates a JSON-format file containing the patient’s data. This file includes all the user’s appointments (Figure 6), with their corresponding dates, hours, and details. It also includes all data on prescriptions, with the medication details, the different intake schedules, and recurrences. Once the file is generated, the smartphone sends it to the assistant.

**Figure 7:**
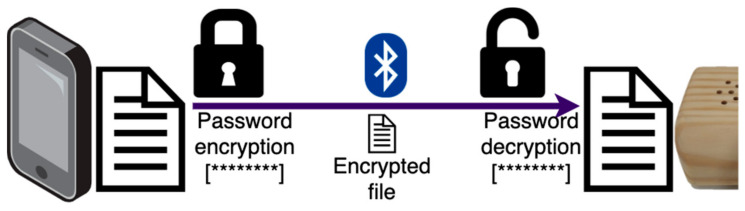
Synchronisation process following symmetric encryption.

Synchronisation is a very sensitive process. It consists on the transmission of the file generated from the smartphone to ACHO, via a Bluetooth connection. Thus, special care has been taken to prevent the possibility of data loss or theft, or malicious attacks that could compromise the user’s medical information. Figure 7 represents how both devices implement security mechanisms based on symmetric encryption—a single password is used to both encrypt and decrypt the file. Furthermore, privacy is guaranteed by the fact that no external entities are involved in data processing and handling, and only the caregiver can access it. As a result, the synchronisation procedure is performed in a secure way.

#### 3.2.2. Voice Assistant

**Figure 8:**
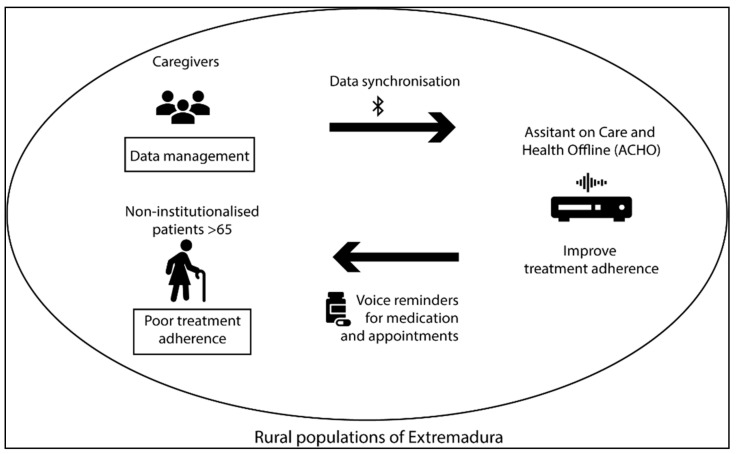
ACHO operational diagram: Bluetooth connection and current functionalities.

The process of synchronisation of the voice assistant with the mobile application finishes when the device receives a data file and starts programming alerts and reminders (Figure 8). As a result of the synchronisation, the voice assistant will automatically and proactively generate notifications corresponding to scheduled events at a certain time and date when the event is due. There are two different kinds of notifications, depending on the event due (i.e., medical appointment, medication).

The medication intake schedule involves a cycle of announcements based on the user’s participation. The device interacts with the user, asking whether the medication has been taken, and repeating the alarm if it does not receive an answer or if this is negative. If the user confirms that the medication has been taken, the device registers the intake as complete. The answers received at this stage are checked, with three possible outcomes: the user answers positively, the user answers negatively, or the user does not answer. Then, Artificial Intelligence technology is used to assess the interaction with the user and the answers provided, and thus establish which treatments are being adhered to and which are not.

### 3.3. Originality of the Proposal

**Figure 9:**
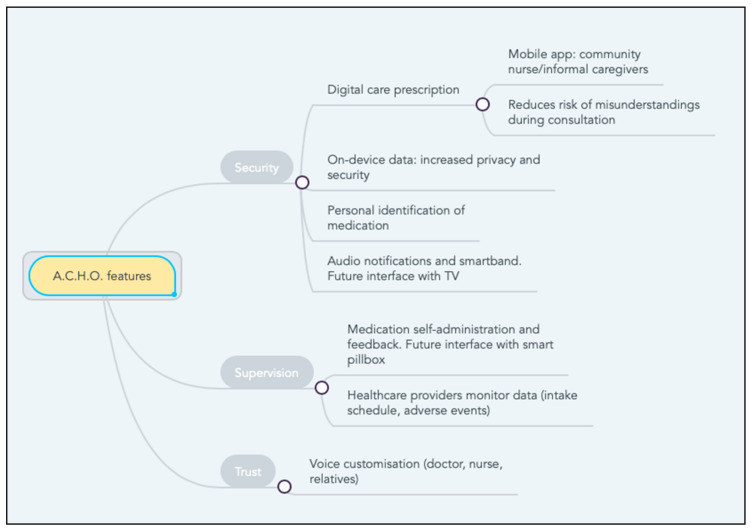
ACHO features.

The originality of this voice assistant is that its functionalities are adapted to the specific requirements of elderly, non-institutionalised, rural populations (Figure 9). It is hoped that ACHO can help improve poor levels of treatment adherence among patients with chronic conditions who live independently in rural areas. We argue that the improvements this system introduces in data privacy and security, doctor–patient relationships, and medication management and supervision can help improve treatment adherence rates.

On the one hand, in the essentially rural region where our study was conducted—with its particular geographic and socio-demographic profile—it is crucial to provide solutions whose use does not require an Internet connection. A large percentage of the population with long-standing illnesses in this region are elderly adults who live in villages without adequate digital communication infrastructures and where Internet coverage is unreliable. Moreover, many of them do not have Internet provision in their homes—a requirement for most widely available voice assistants. By contrast, the fact that ACHO does not require an Internet connection—which could be seen, for some users, as a disadvantage—is in this case a key feature that would enable its full-scale implementation in the houses of many of the patients considered. The use of a mobile application by caregivers, relatives, or healthcare workers facilitates data synchronisation and offers the possibility that community nurses’ home visits can also be used to prescribe “digital care” and to monitor data on medication intake and potential adverse events—all of which would be logged by the voice assistant. Data on medication intake are currently generated by the end-user, but we are working on synchronising this device with a smart pillbox. The introduction of prescriptions directly into the mobile application helps improve safety in medication administration, by helping reduce potential misunderstandings during patient consultation. Moreover, since all data are kept on-device and strict security protocols have been implemented, the possibility of data theft or misuse is also prevented.

Finally, one of the most important features of this voice assistant is the possibility of customising its content through the mobile application. For instance, when a new medication prescription is introduced, the assistant provides the option of re-naming and personalising how it is identified. Many of the participants in our study mentioned their difficulties in recalling the original name of their medication—because it was either difficult to pronounce or to remember. Therefore, many of them used alternative names based on the name of the illness (“the pill for blood pressure”), the colour of the pill (“the red pill”), or the time of the day when it was due (“the one taken at night”). Our mobile application provides the option of customising the name of the different medications, thus helping improve treatment compliance and reducing the risk of the patient getting their medications confused. They also frequently identified the different medications by the colour of the packaging, which can sometimes change. However, another feature of ACHO is that any labels can be easily updated. Therefore, by using the patients’ own recall strategies ACHO facilitates an improved medication administration procedure.

Another feature in the current design is the possibility of customising the voice assistant to use the voice of somebody close to the patient who inspires trust. The voices of their doctors or nurses can be useful in that they inspire authority and confidence in the system, while those of relatives can help reduce feelings of loneliness. During fieldwork, another flaw in the original design was noticed—voice notifications were not always heard. A possibility considered was to relay the notifications through a smartband connected to the assistant, which would provide an alarm when an event was due so that the user could check it. However, our fieldwork revealed a lack of interest in technological innovations among the population studied—which contrasts with the central role that televisions played within their households. Therefore, we are considering ways in which ACHO could interface with their televisions, so reminders could be relayed through video messages.

## 4. Discussion

During the last decade, there has been a growing interest in developing technological applications to improve treatment adherence among chronically ill patients [64,65]. These have ranged from relatively simple systems including telephone calls [66], electronic pillboxes [67], or Short Messages System (SMS) sent to mobile phones [68,69,70,71,72], to more elaborate devices that provide audible [68,69] or audiovisual electronic reminders [73,74]. Other systems explored include the use of Personal Digital Assistants (PDAs) [75] or smartphones. There is a large number of simple applications [76,77,78] that can send alarms to predetermined contacts to let them know whether the user has taken their medication or not [79,80,81], while others can improve communications with healthcare providers [82]. These different technological solutions have improved the rates of treatment adherence [83,84], as well as allowing more secure and regular monitoring of patients [85,86]. A number of studies have noted the importance that some patients attribute to the technological devices that help them remember when their medications are due [87].

Voice assistants such as Alexa, Google Assistant, Siri, and other, less well known systems are of great interest for the scientific healthcare community, due to their easy use, widespread availability, and wide range of functionalities [88]. Their use has opened new possibilities for a wide range of people—including elderly adults who otherwise have no interest or expertise in technological devices. However, data reported about their use seem ambivalent: while a number of studies have argued their efficiency in healthcare due to their use of natural language and ability to simulate human conversations [87,89,90], others have suggested that interactions through these kinds of devices are inconsistent and incomplete [91,92], which can have dangerous consequences. In order to improve these issues, researchers are developing specific skills or applications to help integrate the medical agenda, prescription reminders, and other healthcare monitoring systems into existing devices [93]. For instance, Vidal et al. have used Google Mini’s voice assistant to send reminders about the name, time, and amount of medication that patients need to take. Patients receive a notification for each medication 30 minutes before it is due, again 10 min before, and then when it is due [93]. Our research team has explored similar avenues in the past, through the Remembranza Pills project—a medication management skill developed for Alexa [94]. However, it is difficult to find examples in the scientific literature of voice assistants that can be adapted to specific contexts, despite the growing body of evidence available suggesting that technological solutions need to be user-oriented [95]. A number of studies have argued that adapting technology to the specific needs of elderly populations can significantly increase both performance and acceptance [96]. The voice assistant presented here stems from an opposite idea, situating the users’ needs at the start of the design process, and creating specific solutions adapted to these—for instance, the need to customise the names by which medications are known to patients.

Moreover, all smart speakers currently used by healthcare providers require a constant connection to the Internet [97]. However, and according to data from the Spanish National Institute of Statistics [98], use of the Internet is inversely proportional to age for both male and female users—a trend which is even more marked in rural areas, which frequently lack adequate communication infrastructures. This creates a lack of equal opportunity in the technological solutions available for older, chronically ill adults, depending on whether they live in rural or urban areas [99]. By contrast ACHO, whose use does not require an Internet connection or a minimum level of coverage, is a particularly successful solution that integrates the global trend to develop Ambient-Assisted Living solutions with the contextual fact that many elderly, rural populations lack access to the Internet. Additionally, this feature makes private data handling in ACHO a simpler and more transparent procedure.

Therefore, ACHO provides several improvements and opens up future possibilities. It is clear that in the future, the use of technological solutions will change the way in which healthcare providers deliver care to elderly adults—with a growing use of assistive technology (AT) [100]. According to a survey carried out by *Nuance Healthcare* (2013), eight out of ten healthcare professionals believe AT could help improve patient care and treatment adherence. However, recent studies suggest that one of the main obstacles for the development and implementation of digital care strategies by healthcare providers lies in fact in the deficient digital skills of many healthcare professionals [61]. ACHO could help improve this situation with its simple and intuitive user interface, which enables a rapid learning curve and adaptation—meaning it can be used without prior experience of similar applications [101]. This makes it particularly accessible for elderly patients too, avoiding the usability gap created by other, more complex devices [102,103].

The literature on the topic has underscored the importance of patient motivation for proactively taking action on their health [102]. However, most projects using voice assistants to improve adherence to treatment have failed to address the fact that, because their chosen applications are based on closed platforms, their usability is determined and restricted by the device’s features. Therefore, critical features enabling proactive responses cannot be added on [84], resulting in unidirectional conversations that can only start with the user prompting the device. This is not the case with ACHO, whose proactive behaviour is embedded in its design concept—the device is programmed to initiate interactions with the user. This functionality is a key pillar of active care, since its implementation creates a direct form of communication with users.

Other problems identified as lowering treatment adherence are those associated with patients’ mistakes regarding the medication prescribed, misunderstandings, and lack of efficient communication during medical consultations [104,105]. ACHO can help minimise these kinds of mistakes, since the prescription can be introduced in the application directly by healthcare professionals, and messages identifying different medications can be customised and adapted to the patient’s preferences.

One design limitation is that it is restricted to Android devices, which restricts it to smartphone users with this platform. Information on adherence to the medication schedule can also be unreliable when data are introduced by the patient themselves—the procedure might be affected by involuntary mistakes or forgetfulness. Our fieldwork also revealed problems with voice messages in users with impaired hearing. Therefore, we have identified a future area of development—enabling ACHO to interface with other devices such as smartwatches or smart pillboxes, which can help manage notifications and alerts in a more precise way, while also exploring the novel avenues created by the so-called “Internet of Things” [106].

## 5. Conclusions

The main goal of this article was to introduce the development of a device that is culturally and socially adapted to the capacities of the target audience, as well as to their social trajectory, ideologies, and structural context. ACHO is an innovative technological solution, culturally adapted, whose design is based on a patient-oriented methodological approach. Its ability to interact with users without depending on an Internet connection is one of its main features. Specifically designed for a context of rural, elderly populations among whom comorbidities and polypharmacy are frequent occurrences, its features and functionalities provide a safer medication administration procedure, improved monitoring strategies, and increased patient trust in the prescription process—thus decisively supporting treatment adherence. Design aspects such as the use of voice to interact with the patients (without requiring them to learn or master a new technology), Internet-free functions, and the possibility of customising medication names are features based on the insights gained during the qualitative research stage.

The result of an interdisciplinary effort, ACHO underscores the potential of collaborations between different areas of knowledge and different ways of thinking. The health issues arising from ageing require increasingly innovative practical solutions, and ACHO is an example of how technology can be put at the service of practical healthcare solutions.

## Figures and Tables

**Table 1:** Research techniques.

Research Technique	Empirical Material Obtained
Field diary	Notes taken during fieldwork. Information on context and on additional topics
Participant observation	Observation of everyday activities, particularly regarding medication management (intake schedule, storage, re-naming practices)
Informal conversations	Conducted independently from the interview guide, these can open alternative epistemological windows
Semi-structured interviews	Categories include issues regarding treatment adherence among chronically ill patients

**Table 2:** Example of categories and questions for the interviews.

Categories	Questions
Problems with therapeutic itineraries	Do you have problems when trying to make an appointment? What is the procedure? Is it easy? Why does it take so long/so little, and why do you think that is? Do you have any problems in accessing the doctor’s office? How would you describe your relationship with your doctor?
Treatment and different itineraries	Did you start treating your illness before you were diagnosed? How do you think your health problem can be treated? What previous health problems have you had, and how did you deal with them? How do you feel about the care you have received from your doctor? Tell us about your search for solutions or treatments.
Problems with medication	What medications do you take? Do you always take your medication? Why do you sometimes forget? Do you have any help to remember to take your medication? Do your relatives help you to remember? Does forgetting to take your medication result in health problems?

## Data Availability

The data presented in this study are available on request from the corresponding author. The data are not publicly available due to ethics reasons.
